# RNA-seq Characterization of Sex-Differences in Adipose Tissue of Obesity Affected Patients: Computational Analysis of Differentially Expressed Coding and Non-Coding RNAs

**DOI:** 10.3390/jpm11050352

**Published:** 2021-04-28

**Authors:** Federica Rey, Letizia Messa, Cecilia Pandini, Erika Maghraby, Bianca Barzaghini, Maria Garofalo, Giancarlo Micheletto, Manuela Teresa Raimondi, Simona Bertoli, Cristina Cereda, Gian Vincenzo Zuccotti, Raffaella Cancello, Stephana Carelli

**Affiliations:** 1 Department of Biomedical and Clinical Sciences “L. Sacco”, School of Medicine, University of Milan, Via G.B. Grassi 74, 20157 Milan, Italy; federica.rey@unimi.it (F.R.); erika.maghraby@studenti.unimi.it (E.M.); gianvincenzo.zuccotti@unimi.it (G.V.Z.); 2 Paediatric Clinical Research Center Fondazione “Romeo ed Enrica Invernizzi”, University of Milan, Via G.B. Grassi 74, 20157 Milan, Italy; 3 Department of Chemistry, Materials and Chemical Engineering “Giulio Natta”, Politecnico di Milano, Piazza Leonardo da Vinci 32, 20133 Milan, Italy; letizia.messa@mail.polimi.it (L.M.); bianca.barzaghini@polimi.it (B.B.); manuela.raimondi@polimi.it (M.T.R.); 4 Genomic and Post-Genomic Center, IRCCS Mondino Foundation, Via Mondino 2, 27100 Pavia, Italy; cecilia.pandini@mondino.it (C.P.); maria.garofalo@mondino.it (M.G.); cristina.cereda@mondino.it (C.C.); 5 Department of Pathophysiology and Transplantation, INCO and Department of General Surgery, Istituto Clinico Sant’Ambrogio, University of Milan, Via Francesco Sforza 35, 20122 Milan, Italy; giancarlo.micheletto@unimi.it; 6 Obesity Unit—Laboratory of Nutrition and Obesity Research, Department of Endocrine and Metabolic Diseases, IRCCS Istituto Auxologico Italiano, Via Ariosto 9, 20145 Milan, Italy; simona.bertoli@unimi.it (S.B.); r.cancello@auxologico.it (R.C.); 7 International Center for the Assessment of Nutritional Status (ICANS), Department of Food, Environmental and Nutritional Sciences (DeFENS), University of Milan, Via Celoria 2, 20133 Milan, Italy; 8 Department of Pediatrics, Children’s Hospital “V. Buzzi”, Via Lodovico Castelvetro 32, 20154 Milano, Italy

**Keywords:** obesity, sex, RNA-seq, lncRNAs, gene expression, metabolic diseases, computational prediction, bioinformatics, transcriptome, gender differences

## Abstract

Obesity is a multifactorial disease presenting sex-related differences including adipocyte functions, sex hormone effects, genetics, and metabolic inflammation. These can influence individuals’ risk for metabolic dysfunctions, with an urgent need to perform sex-based analysis to improve prevention, treatment, and rehabilitation programs. This research work is aimed at characterizing the transcriptional differences present in subcutaneous adipose tissue (SAT) of five obesity affected men versus five obesity affected women, with an additional focus on the role of long non-coding RNAs. Through RNA-sequencing, we highlighted the presence of both coding and non-coding differentially expressed RNAs, and with numerous computational analyses we identified the processes in which these genes are implicated, along with their role in co-morbidities development. We report 51 differentially expressed transcripts, 32 of which were coding genes and 19 were non-coding. Using the WGCNA R package (Weighted Correlation Network Analysis, version 1.70-3), we describe the interactions between coding and non-coding RNAs, and the non-coding RNAs association with the insurgence of specific diseases, such as cancer development, neurodegenerative diseases, and schizophrenia. In conclusion, our work highlights a specific gender sex-related transcriptional signature in the SAT of obesity affected patients.

## 1. Introduction

Obesity is a multifactorial disease that, alongside overweight, affects over 1.9 billion adults worldwide [1]. The prevalence of obesity is on the rise in many countries throughout the world; it has tripled since the 1960s, and obesity can thus currently be considered an epidemic and a major health issues [2]. Epidemiologic studies demonstrate that overweight and obesity in both men and women are associated with various aberrations including increased propensity to diabetes, cancer, and lipid profile [3,4]. Even so, there are sex-related differences in obesity including adipocyte functions, sex hormone effects, genetics, and metabolic inflammation issue, which should be considered when studying an individuals’ risk for metabolic dysfunctions [3,5,6]. According to the World Health Organization, sex “refers to the biological and physiological characteristics that define men and women,” whereas gender “refers to the socially constructed roles, behaviors, activities, and attributes that a given society considers appropriate for men and women” [7]. This knowledge is valuable in devising preventative measures and treatments for both men and women. Sex differences have frequently underestimated the consequences on the daily practice of medicine and in the developing specific therapeutic approaches. In fact, recent pharmacovigilance studies on large populations indicate that the risk of drug side effects in women is about 40% higher than in men during clinical trials [8]. Despite this, relatively few studies have investigated these aspects in obesity research. There is thus an urgent need to investigate sex-based analyses to improve prevention and treatment of this pathology [8,9,10]. 

Although men and women can both become obese, the incidence and health consequences differ between the sexes, starting from early childhood [10,11]. Moreover, they differ in genetics and in many biological processes such as fat deposition, its mobilization, and its use as a metabolic fuel and the consequences of both excess and insufficient fat stores [12,13,14]. Indeed, women usually have more subcutaneous adipose tissue (SAT) in the gluteal-femoral body area while men have more visceral adipose tissue (VAT) around the abdominal organs, resulting in two different body shapes. Despite the extensive knowledge of adipose tissue distribution, only a few studies have focused on how sex contributes to differences in adipose metabolism [15]. One work including a large cohort of 359,387 participants from nine countries confirmed that there is an association of BMI and waist-to-hip ratio (WHR), with slight differences between the sexes [16]. To support genetic differences between sexes in obesity, a meta-analysis of genome-wide association data from 38,580 individuals, followed by large-scale replication (in up to 70,689 individuals) has identified two loci (TFAP2B and MSRA) and a further locus, near LYPLAL1, suggesting gender-specific relationships with WHR and overall adiposity [17]. An additional meta-analysis of 32 genome-wide association studies for WHR adjusted for BMI identified 13 new loci in or near RSPO3, VEGFA, TBX15-WARS2, NFE2L3, GRB14, DNM3-PIGC, ITPR2-SSPN, LY86, HOXC13, ADAMTS9, ZNRF3-KREMEN1, NISCH-STAB1 and CPEB4 that exhibited a stronger effect on WHR in women than men, providing further evidence for multiple loci that modulate body fat distribution and reveal strong gene-by-sex interactions [18].

Even with an increasing number of genetic differences between women, these do not account for all observed phenotypes. Linkage analysis, especially in twins, confirmed that genetics explains 40–80% of the variance in body mass index (BMI) and in risk of obesity [19]. Indeed, there is a need to increase current knowledge, extending research efforts not only on the role of genetics, but also of epigenetics and transcriptional regulation. To investigate whether robust sex differences exist in adipose tissue gene expression, one very recent work revealed 162 differentially expressed genes, with those with an increased expression in men being associated with sex-hormone receptors, while genes that were elevated in women being implicated in oxidative phosphorylation and adipogenesis [20]. Moreover, the role of the non-coding component of the genome could play a significant role in determining differences in BMI and disease development [21]. Previously classified as “junk DNA”, non-coding RNAs (ncRNAs) are a class of molecules with multiple functions in both health and disease [22,23,24,25,26]. They can be classified into subfamilies according to their size, the main ones being small-size microRNAs (miRNA/miR, <200 nucleotides) and long non-coding RNAs (lncRNAs, >200 nucleotides) [24]. The role of miRNAs in genetic sex differences in adipose tissue has recently been studied by performing high-throughput miRNA sequencing in gonadal fat depots of the Four Core Genotypes mouse model, which allowed us to assess the independent effects of gonadal type (male vs. female) and sex chromosome complement (XX vs. XY) on miRNA expression profiles [27]. Moreover, gender and obesity specific microRNAs have been found to be altered in the adipose tissue from lean and obese pigs [28].

As transcriptional regulators, lncRNAs represent emerging targets to study adipocytes development in obese patients [29,30]. Recent studies have addressed the role of lncRNAs in the network governing adipogenesis and adipocytes biology. Some lncRNAs play a key role in these phenomena by interacting with different transcription factors, such as PGC-1alpha, EBF2, ZBTB7B, ZFP516, and PRDM16 [31]. Moreover, lncRNAs could play a remarkable role in explaining gender differences. Indeed, the most notable contribution of lncRNA to sex differences is provided by the lncRNA XIST, encoded on the chromosome X [32,33,34]. Sexual dysmorphisms correlated with lncRNAs have been found for the brain, in male sex determination, and even cardiovascular diseases, but no specific evidence has to this day been reported for the adipose tissue and obesity [35,36,37]. To this end, this research work is aimed at the characterization the transcriptional differences present in the SAT of men and women, with an additional focus on the role of lncRNAs.

## 2. Materials and Methods

### 2.1. Adult Human Adipose Tissue Collection, Isolation and Differentiation

The present study is in accordance with the Declaration of Helsinki, and it was approved by the Ethical Committee of IRCCS Istituto Auxologico Italiano (Ethical Committee approval code #2020_10_20_04). A signed informed consent was obtained from each enrolled patient for tissue sampling. Biopsies of SAT were collected from 5 obese women (OBF; age 41 ± 12.5 years, BMI 38.2 ± 4.6 kg/m^2^) and 5 obese men (OBM; age 42.4 ± 6.58 years, BMI 36.9 ± 3.5 kg/m^2^), and their clinical features are reported in Table S1. Surgical biopsies of whole abdominal SAT were collected pre-operatively from obese patients during bariatric surgery procedures and from normal weight patients before aesthetic plastic surgery or abdominal surgery for non-inflammatory diseases. Each collected biopsy was weighed and stored in 1 mL of DMEM (Invitrogen Corporation, Jefferson City, MO, USA) supplemented with 2.5% Bovine Serum Albumin (Sigma, St. Louis, MO, USA) per gram of collected tissue. The biopsy was immediately transferred to the laboratory and processed. A fragment of the whole adipose tissue biopsy was immediately frozen in liquid nitrogen for RNA extraction.

### 2.2. SAT RNA Extraction

Approximately 500 mg of frozen SAT was homogenized in RLT buffer (Qiagen, Hilden, Germany). RNA from SAT was extracted using the RNeasy Mini Kit (Qiagen) according to the manufacturer protocol, and samples were then treated with the RNase-Free DNase Set (Qiagen). Concentration and quality of the extracted RNA were determined by the NanoDrop ND-1000 spectrophotometer (NanoDrop Technologies, Wilmington, DE, USA) and RNA integrity verified by gel-electrophoresis.

### 2.3. Libraries Preparation for RNA-seq and Bioinformatic Data Analysis

RNA-seq libraries were prepared with the CORALL Total RNA-Seq Library Prep Kit (Lexogen, Vienna, Austria) using 150 ng total RNAs. The RiboCop rRNA Depletion Kit (Lexogen, Vienna, Austria) was used to remove rRNA. Qualities of sequencing libraries were assessed with D1000 ScreenTape Assay using the 4200 TapeStation System (Agilent, Santa Clara, CA, USA) and quantified with Qubit™ dsDNA HS Assay Kit (Invitrogen, Carlsbad, CA, USA). RNA processing was carried out using Illumina NextSeq 500 Sequencing. FastQ files were generated via llumina bcl2fastq2 (Version 2.17.1.14—https://support.illumina.com/downloads/bcl2fastq-conversion-software-v2-20.html, last accessed on 15 February 2021), starting from raw sequencing reads produced by the Illumina NextSeq sequencer. The quality of individual sequences were evaluated using FastQC software (version 0.11.9; https://www.bioinformatics.babraham.ac.uk/projects/download.html#fastqc accessed on 26 March 2021) after adapter trimming with cutadapt software. Gene and transcript intensities were computed using STAR/RSEM software (version 2.7; https://code.google.com/archive/p/rna-star/ accessed on 26 March 2021) [38] using Gencode Release 27 (GRCh38) as a reference, using the “-strandness forward” option. Transcript abundance was obtained using the BlueBee^®^ Genomics Platform (Lexogen). Differential expression analysis for mRNA was performed using R package DESeq2 [39]. Genes were considered differentially expressed and retained for further analysis with |log2(men SAT/women SAT)| ≥ 1 and an FDR ≤ 0.1. We imposed minimum |log_2_FC| of 1 and an FDR lower than 0.1 as thresholds to differentially expressed genes. The raw data obtained from the RNA-seq analysis was deposited on the Gene Expression Omnibus repository with the accession number GSE166047.

### 2.4. Pathway Analysis

Gene enrichment analysis was performed on coding genes using g:Profiler, ranking terms according to their fold change and using a Bonferroni-Hochberg FDR of 0.05 as the threshold [40]. The R software was used to generate heatmaps (heatmap.2 function from the R ggplots package), PCA plots (prcomp function from the R ggplots package), Volcano plots [41], Dotplot graphs (ggplot2 library), and Pathview graphs (Pathview library [42]). The NDEx plugin [43] was used to perform analysis regarding the tissue expression, subcellular localization, and protein interaction of the differentially expressed genes [44]. The iRegulon plugin was used to identify the transcription factors possibly interacting with the DE RNAs and visualized using Cytoscape [45].

### 2.5. Coding and ncRNAs Co-Expression Analysis

Coding RNAs’ co-expression with ncRNAs was performed using the weighted gene co-expression network analysis (WGCNA) R package (https://CRAN.R-project.org/package=WGCNA, last accessed on 25 March 2021) [46]. The soft thresholding power was chosen considering the criterion of approximate scale-free topology. Network nodes represent gene expression profiles, while undirected edges values are the pairwise correlations between gene expressions. Cytoscape software was used for network import and visualization.

### 2.6. Correlation Analyses

For correlation analyses, the DE RNAs were correlated with anthropometrical parameters corresponding to specific patients. For each gene, the raw counts were normalized on the raw counts of EEF2 (Eukaryotic Translation Elongation Factor 2), identified as stable housekeeping gene from the Housekeeping and Reference Transcript Atlas [47]. Prism 8 software (GraphPad Software Inc., La Jolla, CA, USA) was used for statistical analysis, assuming a *p* value less than 0.05 as the limit of significance.

### 2.7. RNA Extraction and Real-Time PCR

Real-Time PCR was performed with the CFX Connect Real-Time RT-PCR System (Bio-Rad, Hercules, CA, USA) using the SsoAdvancedTM Universal SYBR^®^ Green Supermix (Bio-Rad, Hercules, CA, USA) as dye. Primers were designed with NCBI’s Primer-BLAST tool, and they are reported in Table S2. Gene expression was calculated using the 2^−ΔΔCt^ method, and 18S was used as an endogenous control. Data were expressed as mean ± SEM. The statistical analysis was performed with Student’s *t*-test. Prism 7 software (GraphPad Software Inc., La Jolla, CA, USA) was used, assuming a *p*-value less than 0.05 as the limit of significance.

## 3. Results

### 3.1. Deep Sequencing of RNAs Expression Profiles in SAT from Obese Women and Men Reveals Transcriptional Differences

**Figure 1:**
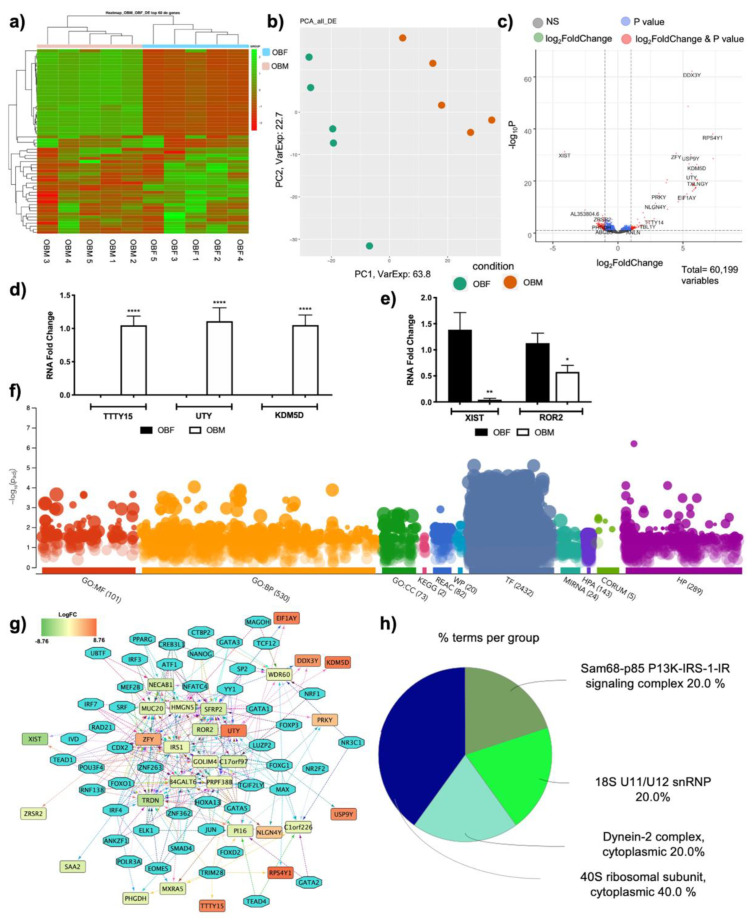
Transcriptome analysis highlights different expression profiles in the SAT of obese men versus women: (**a**) Heatmap analysis of differentially expressed genes (DE RNAs) in obese men (OBM) versus obese women (OBF). (**b**) Principal Component Analysis (PCA) of DE RNAs in OBM versus OBF. (**c**) Volcano plot showing DE RNAs between OBM and OBF. (**d**) mRNA expression levels of genes with a positive fold change were evaluated by Real-Time PCR in 11 OBF vs. 7 OBM. Data are expressed as mean ± SEM. **** *p* <0.0001 vs. OBF. (**e**) mRNA expression levels of genes with a negative fold change were evaluated by Real-Time PCR in 11 OBF vs. 7 OBM. Data are expressed as mean ± SEM. * *p* < 0.05, ** *p* < 0.01 vs. OBF. (**f**) DE RNAs were subjected to functional enrichment analysis via the g:Profiler web tool, ranking the genes in term of their absolute fold change and considering a Bonferroni–Hochberg FDR of 0.05 as significance threshold. A significant dysregulation was observed in all categories analyzed, which include Gene Ontology (GO), with 101 significant pathways for molecular function (MF), 530 significant pathways for biological processes (BP), and 73 significant pathways for cellular component (CC), pathway analyses that highlighted 2 significant pathways for KEGG, 82 significant pathways for Reactome, and 20 significant pathways for WikiPathways, along with 2432 regulatory motifs in DNA (TF), 24 genes targeted by miRNAs (MIRNA), protein expression (HPA, 143) implications for protein complexes with CORUM (5), and implications for human phenotypes (HP, 289). (**g**) Regulon analysis of DE RNAs reports the identification of TFs binding to specific motifs present in DE RNAs. TFs are reported as white-filled hexagon shapes, whereas DE RNAs are reports as color-filled rectangles. The color of DE RNAs indicates the respective log_2_FC. (**h**) CORUM protein complexes analysis displayed as ClueGO graph. Each pie segment refers to the % of terms present per group.

This work was aimed at characterizing the implication of sex differences in the subcutaneous adipose tissue (SAT) of obesity affected patients, fully characterizing the underlying molecular signature that leads to a different disease profile in obese men or women. Heatmap analysis of the DE RNAs showed different expression profiles (Figure 1a), and the same can be concluded when looking at the PCA representation (Figure 1b). Indeed, the two categories tend to group together, revealing a transcriptional dysregulation. The volcano plot reports the DE RNAs (Figure 1c), and specifically 51 DE RNAs were identified, 70% of which were coding genes and 30% of which were non-coding DE RNAs (Table 1).

**Figure 2:**
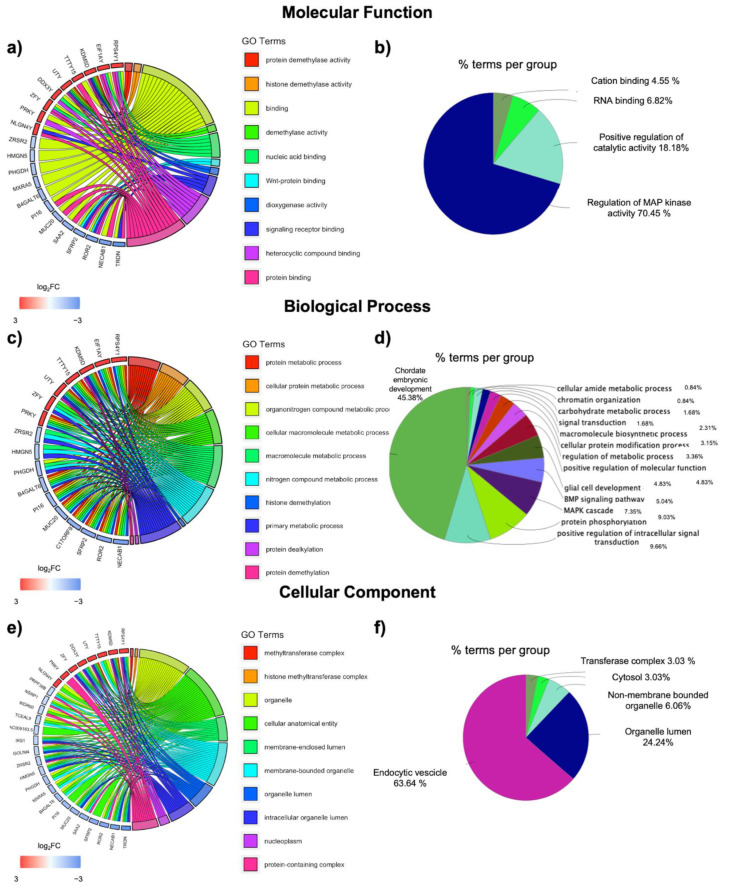
Gene Ontology in obese men vs. women. Gene ontology was performed for molecular function and displayed as a GO Chord (**a**) or a ClueGO Graph (**b**), for biological processes it is displayed as a GO Chord (**c**) or a ClueGO Graph (**d**), and for cellular component it is displayed as a GO Chord (**e**) or a ClueGO Graph (**f**). For the GO Chord graphs, on the right, are shown the top 10 significant GO terms for the cellular component, whereas on the left the corresponding genes are ordered according to log_2_FC. Segments connect each term to the respective involved gene. For the ClueGO graphs, each pie segment refers to the % of terms present per group.

The list of coding DE RNAs, ranked by their FC, is reported in Table 2. Specifically, it is possible to notice genes involved in transcriptional control, including ribosomal proteins, translation initiation factors and activators, demethylases, regulators of proteins turnover, and even splicing modulators, suggesting a profound transcriptional dysregulation. Via Real-Time PCR, we validated the expression of 3 genes with increased expression in males (*TTTY15, UTY*, and *KDM5D*, Figure 1d) and 2 genes with decreased expression (*XIST, ROR2*, Figure 1e) in an independent cohort of SAT samples obtained from 11 females affected by obesity and 7 males affected by obesity, supporting the evidence of the RNA-seq analysis.

The DE RNAs transcripts for OB-M vs. OB-F (|Log_2_FC| ≥ 1) were subjected to pathways analysis using g:Profiler [40], ranking the genes in term of their |FC|. A significant dysregulation was observed in all categories analyzed, which included gene ontology (GO), with 101 significant pathways for molecular function (MF), 530 significant pathways for biological processes (BP), and 73 significant pathways for cellular component (CC), pathway analyses, which highlighted 2 significant pathways for KEGG, 82 significant pathways for Reactome, and 20 significant pathways for WikiPathways, along with 2432 regulatory motifs in DNA (TF), 24 genes targeted by miRNAs (MIRNA), and implications in protein databases such as the human protein atlas (HPA, 143), CORUM (5), and implications for human phenotypes (HP, 289) (Figure 1d, Table S3). The iRegulon plugin was used to identify the transcription factors possibly interacting with the DE RNAs (Figure 1e). Specifically, 47 transcription factors were identified as interacting with 25 DE RNAs. Moreover, the 5 CORUM protein binding complexes formed by the DE RNAs were represented, and these were the cytoplasmic 40S ribosomal subunit, the cytoplasmic dynein-2 complex, the Sam-68-p85 P13K-IRS-1-IR signaling complex, and 18S U11/U12 snRNP (Figure 1f). 

### 3.2. GO Terms Enrichment Shows Numerous Differences in Genes Pertaining Specific MF, BP, and CC

The GO terms analysis in MF highlighted 101 significant pathways, and the top 10 are displayed as a GO Chord graph in Figure 2a. Interestingly, the highest perturbation seems to be due to the increased expression of two demethylating enzymes: KDM5D and UTY. Indeed, amongst the perturbated processes it is possible to notice “protein demethylase activity”, “histone demethylase activity”, and “demethylase activity”. The pathways involving the highest number of genes appears to be “binding”, further subdivided into specific binding of nucleic acid, Wnt-protein, signaling receptor, heterocyclic compound, and protein binding. ClueGO MF analysis highlights as the top significant pathways the “regulation of MAPK activity” along with “cation binding” and “RNA binding and catalytic activity” (Figure 2b). For GO BP analysis, 530 significant terms emerged, and again the top 10 are displayed as a GO Chord graph in Figure 2c. Most pathways involve metabolic processes, interesting in the obesogenic context, including terms such as “protein metabolic process”, “cellular protein metabolic process”, “organonitrogen compound metabolic process”, “cellular macromolecule metabolic process”, “macromolecule metabolic process”, “nitrogen compound metabolic process”, and “primary metabolic process”. Protein modifications are also represented, with the rest of the top significant terms pertaining “protein dealkylation”, “protein demethylation”, and “histone demethylation”. ClueGO analysis highlights implication for genes in chordae embryonic development, along with processes already displayed as GO Chord and, interestingly, chromatin organization and specific pathways such as that involving BMP or the MAPK cascade (Figure 2d). Lastly, 73 significant terms emerged in the GO CC analysis, and the top 10 are displayed as a GO Chord graph in Figure 2e. Interestingly, a strong implication is given to the nucleoplasm along with chromatin remodeling complexes such as the “methyltransferase complex” and the “histone methyltransferase complex”. Another compartment seems to be involved in the membrane, with terms such as “membrane-enclosed lumen” and “membrane-bounded organelle”. ClueGO also reports a strong implication for the “endocytic vesicles and the organelles lumen” (Figure 2f). 

### 3.3. Pathway Analysis of DE RNAs Highlights Specific Processes-Involvement

**Figure 3:**
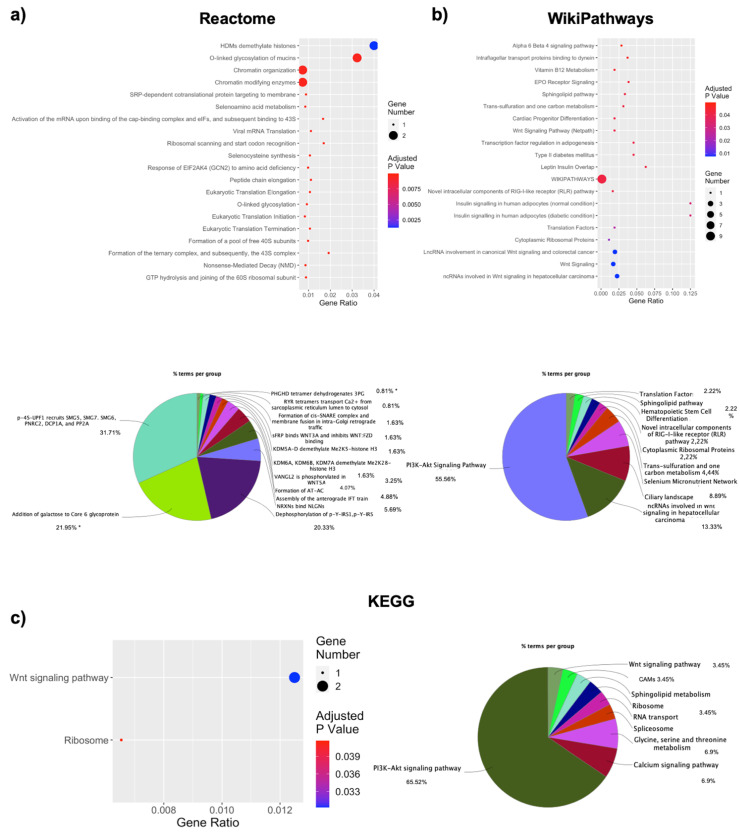
Pathways analysis shows implicated processes in OBM vs. OBF. Pathway analysis of significant processes in Reactome (**a**), WikiPathways (**b**), and KEGG database (**c**), represented as dotplot and ClueGO graphs. For dotplots, the y-axis represents the name of the pathway, the x-axis represents the gene ratio, dot size represents the number of different genes, and the color indicates the adjusted *p*-value. For ClueGO graphs, each pie segment refers to the % of terms present per group and * *p* < 0.05.

DE RNAs were subjected to pathway analysis in three well renowned databases: Reactome, WikiPathways, and KEGG; g:Profiler analysis highlighted 82 significant Reactome pathways, reported in Table S3. The top 20 pathways, ranked by their significance, are reported in Figure 3a. Interestingly, again epigenetic and transcriptional regulation seems to be the most represented terms. Indeed, chromatin remodeling is represented by “HDMs demethylase histones”, “chromatin organization”, and “chromatin modifying enzymes”, whereas numerous steps of protein synthesis, including all the steps of translation, are involved. ClueGO analysis also highlights the implication of multiple demethylase activities, with more pathways pertaining to specific molecules’ involvement and even metabolic processes pertaining, for example, to glucidic metabolism (Figure 3a). Twenty pathways were found after WikiPathways analysis, and these included, again, transcription and translational pathways, along with ribosomal subunits and even an involvement for non-coding RNAs (Figure 3b). The Wnt signaling pathway is represented multiple times, along with insulin signaling and type 2 diabetes. The non-coding and translational dysregulations are also represented after ClueGO analysis, and a strong involvement of the PI3K-Akt signaling pathway is also reported (Figure 3b). KEGG analysis with g:Profiler highlighted two significant pathways, also previously mentioned, which are the Wnt signaling pathway and ribosome involvement (Figure 3c). ClueGO KEGG analysis again highlighted the highest involvement of the PI3K-Akt signaling pathway but also implicated ribosome, RNA transport, and spliceosome as processes that could impact gene expression (Figure 3c). 

### 3.4. Characteristics of DE RNAs in SAT of Men versus Women: Interaction, Tissue Expression and Cellular Localization

**Figure 4:**
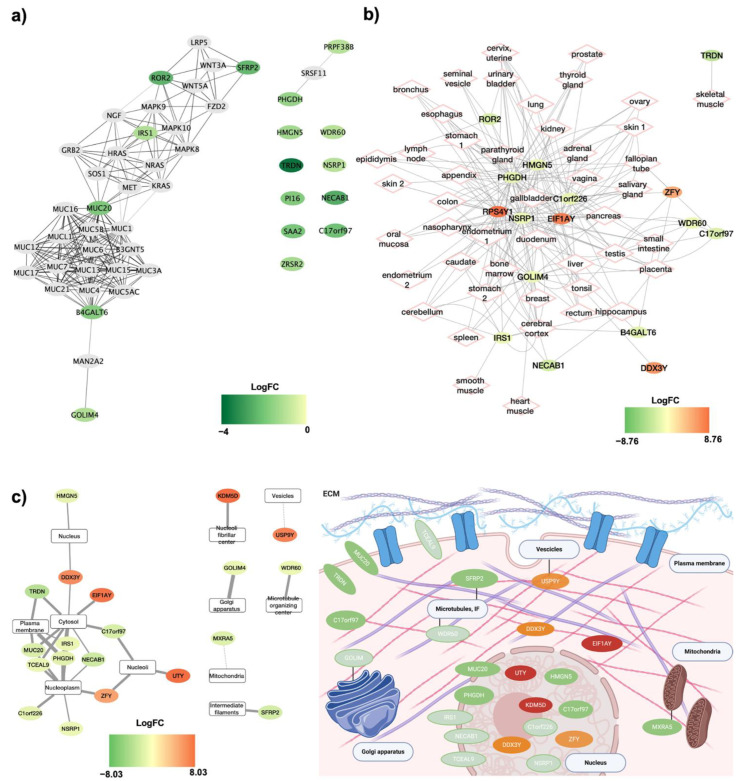
Characterization of DE RNAs interaction, tissue expression, and subcellular localization: (**a**) STRING protein network interaction as obtained through the NDEx plugin of Cytoscape. The DE RNAs are color-filled, and the color indicates the respective log_2_FC. Grey ellipses are the interacting proteins. (**b**) Tissue expression of DE RNAs as obtained through the NDEx plugin of Cytoscape. The DE RNAs are color-filled, and the color indicates the respective log_2_FC. (**c**) Subcellular localization of DE RNAs as obtained through the NDEx plugin of Cytoscape. The DE RNAs are color-filled, and the color indicates the respective log_2_FC. The DE RNAs are also localized using a graphical representation, made with Biorender.com.

The coding DE RNAs were comprehensively characterized for their interaction, tissue expression, and subcellular localization (Figure 4). To do so, the STRING database was used to construct an interaction network of DE RNAs, where the nodes are proteins and the edges represent the predicted functional associations. It is possible to see that the proteins belong to three main networks, with MUC20 being an inter-player amongst two of them (Figure 4a). With the NDEx database, it was also possible to investigate the genes expression and cellular localization. The DE RNAs in the SAT of obese men are also expressed in a high number of other tissues. These also include neural specific tissues such as the hippocampus, the cerebellum, and the cerebral cortex; tissues implicated in the genitourinary apparatus such as the fallopian tube, the endometrium, the urinary bladder, and the seminal vesicle etc.; and also the muscle tissue (e.g., skeletal muscle, heart muscle) (Figure 4b). Moreover, in Figure 4c it is possible to observe the known subcellular localization of the DE RNAs. A high number of variable organelles emerge, and it is possible to suggest that the cells of the SAT present with ubiquitary differential expression, in the nucleus as well as the cytoplasm, the mitochondria, and the cytoskeleton, indicating a profound alteration in all cellular functions.

### 3.5. Analysis of Differential Expression in Relation to Diseases-Development: Implications for Sex Differences in Secondary Co-Morbidities Development

**Figure 5:**
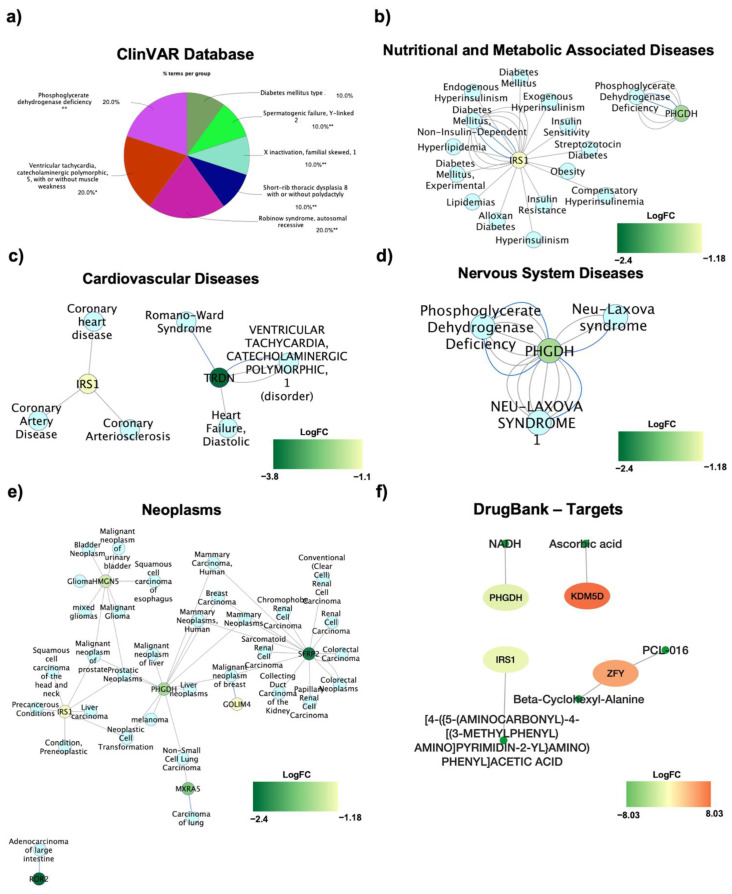
Susceptibility to co-morbidities development in OBM vs. OBF: (**a**) ClinVar database disease implications are reported as a ClueGO graph, where each pie segment refers to the % of terms present per group; * *p* < 0.05 and ** *p* < 0.01. The DisGENET database was used to identify the DE RNAs correlation with (**b**) Nutritional and Metabolic Associated Diseases, (**c**) Cardiovascular Diseases, (**d**) Nervous System Diseases, and (**e**) Neoplasms. (**f**) The NDEx Cytoscape plugin through DrugBank database allowed the identification of potential drug targets. The DE RNAs are color-filled, and the color indicates the respective log_2_FC.

In order to investigate the potential secondary diseases that could arise in either men or women, the ClinVar database was used. This highlighted, as it is to be expected, diseases associated with the X or Y chromosome (spermatogenic failure, Y-linked or X inactivation, familial skewed), but, interestingly, also metabolic diseases such as diabetes mellitus type 2 and phosphoglycerate dehydrogenase deficiency (Figure 5a). This suggests that the genetic signature found here could be responsible for the development of secondary metabolic complications, and indeed, heart failure also emerges (ventricular tachycardia). The DisGENET database was used to highlight the specific genes’ curated evidence, linking them to disease conditions, subdivided by disease category. Specifically, IRS1 and PHGDH emerge as the DE RNAs causative of nutritional and metabolic associated diseases, as they are extremely relevant and characterized in diabetes, lipidemia, obesity (IRS1), and phosphoglycerate dehydrogenase deficiency (PHGDH) (Figure 5b). IRS1 is also involved in the development of coronary heart diseases, whilst the TRDN gene is involved in other forms of heart failure, ventricular tachycardia, and Romano-Ward Syndrome (Figure 5c). The PHGDH is also responsible for the development of neurological genetic Neu-Laxova Syndrome (Figure 5d). Lastly, some genes (*HMGN5, IRS1, PHGDH, MXRA5, GOLIM4,* and *SFRP2*) are associated with the development of multiple cancer types (Figure 5e). Interestingly, all the discussed genes present a decreased expression in men for susceptibility genes, and it could thus be speculated that women patients could be more prone to developing associated pathologies, but of course each gene should be considered for its specific correlation with the analyzed condition. As gender medicine is becoming of crucial relevance, a focus was also given to the potential drugs selectively targeting DE RNAs (Figure 5f). Indeed, four specific genes that could be targeted emerged, and these are *PHGDH, KDM5D, IRS1,* and *ZFY.*

### 3.6. Role of Non-Coding RNAs in Gender Imbalace in Obesity: Interaction with DE RNAs and Correlated Functions

**Figure 6:**
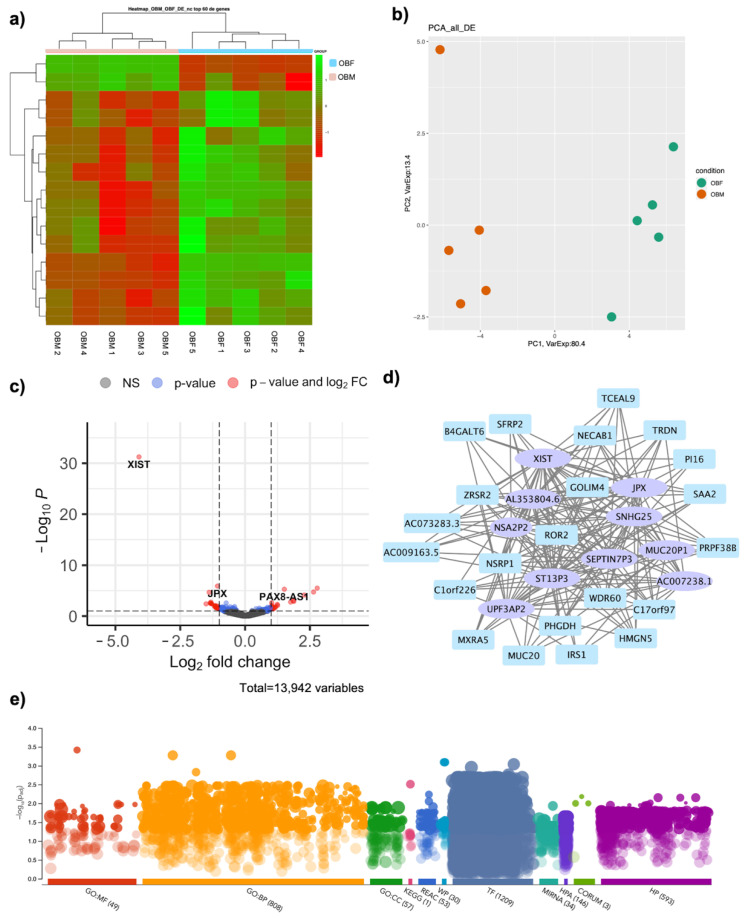
Implications for ncRNAs in DE RNAs in OBM vs. OBF: (**a**) Heatmap analysis of nc DE RNAs in OBM versus OBF. (**b**) PCA of nc DE RNAs in OBM versus OBF. (**c**) Volcano plot showing nc DE RNAs between OBM and OBF. (**d**) WGCNA Co-interaction network between coding and ncRNAs. Coding genes are displayed as light blue rectangles whereas nc DE RNAs are represented as purple ellipses. (**e**) DE RNAs connected to nc DE RNAs were subjected to functional enrichment analysis via the g:Profiler web tool, ranking the genes in term of their absolute FC and considering a Bonferroni–Hochberg FDR of 0.05 as significance threshold. Significant terms were found in all categories analyzed, which include gene ontology (GO), with 49 significant pathways for molecular function (MF), 808 significant pathways for biological processes (BP), and 57 significant pathways for cellular component (CC) pathway analyses, which highlighted 1 significant pathways for KEGG, 53 significant pathways for Reactome, and 30 significant pathways for WikiPathways, along with 1209 regulatory motifs in DNA (TF), 34 genes targeted by miRNAs (MIRNA), protein expression (HPA, 146) implications for protein complexes with CORUM (3), and implications for human phenotypes (HP, 593).

As lncRNAs are becoming more and more relevant in the context of sex-biology and obesity, a special focus was given to the non-coding DE RNAs, as these could bridge the void in the present knowledge concerning the role of lncRNAs in sex-specific obesity. Indeed, non-coding DE RNAs also present diversifies expression profiles, as shown in Figure 6a with Heatmap visualization and in Figure 6b with PCA clustering. The volcano plot reports the non-coding DE RNAs (Figure 6c), and indeed, 19 non-coding RNAs emerge as differentially expressed, and they are thus reported in Table 3. The possible lncRNAs’ implication in diseases was analyzed using the LncRNADiseasev2.0 database [48] to investigate their implication in possible comorbidities development. AC10889.1, TTTY15, XIST, AC006157.1, PAX8-AS1, SNHG25, and JPX were mainly involved in cancer development. Interestingly, apart from AC10889.1 and AC006157.1, the other lncRNAs were implicated, not only in cancer, but also in neurodegenerative diseases (Alzheimer’s disease and Huntington’s disease) and schizophrenia, suggesting they could contribute to co-morbidity development.

A co-interaction network was built, highlighting all the ncRNAs that interact with coding genes (Figure 6d). The network highlights how 10 out of the 15 ncRNAs interact with 22 coding DE RNAs. This suggest that lncRNAs could exert their functions through a modulation of these interacting coding DE RNAs. In order to see in which processes these lncRNAs could be implicated, the functional enrichment of the DE RNAs specifically associated with lncRNAs was analyzed to identify the specific ontology and pathway implication. Indeed, significant terms were found in all categories analyzed, which include gene ontology (GO), with 49 significant pathways for molecular function (MF), 808 significant pathways for biological processes (BP), and 57 significant pathways for cellular component (CC) pathway analyses, which highlighted 1 significant pathways for KEGG, 53 significant pathways for Reactome, and 30 significant pathways for WikiPathways, along with 1209 regulatory motifs in DNA (TF), 34 genes targeted by miRNAs (MIRNA), and implications in protein databases such as the human protein atlas (HPA, 146), CORUM (3), and implications for human phenotypes (HP, 593) (Figure 6e, Table S4).

**Figure 7:**
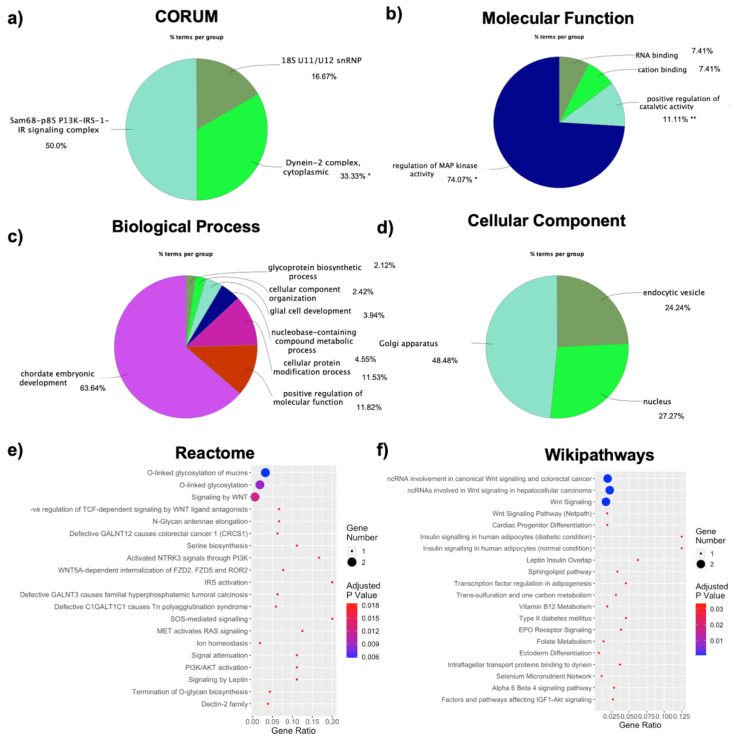
Characterization of enrichment in ncRNAs-related DE RNAs: (**a**) CORUM protein complexes analysis and GO enrichment analyses for (**b**) molecular function, (**c**) biological processes, and (**d**) cellular component, displayed as ClueGO graphs. Each pie segment refers to the % of terms present per group; * *p* < 0.05 and ** *p* < 0.01. Pathways analysis with Reactome (**e**) and Wikipathways (**f**) databases, represented as a dotplot, where the y-axis represents the name of the pathway, the x-axis represents the gene ratio, dot size represents the number of genes, and the color indicates the adjusted *p*-value.

We next specifically investigated these categories and found that the 3 CORUM terms concerned the Sam-68-p85 P13K-IRS-1-IR signaling complex, the cytoplasmic dynein-2 complex, and 18S U11/U12 snRNP (Figure 7a). The GO terms analysis in MF highlighted 49 significant pathways, and the results are displayed as pie graph in Figure 7b. ClueGO MF analysis highlights MAPK activity with 74.07% terms per group, along with catalytic activity with 11.11% terms per group and cation binding and RNA binding with 7.41% terms per group (Figure 2b). Interestingly, the highest perturbation seems to be due to the activity of MAP kinases, which are involved in the regulation of both normal and pathological adipogenesis [49]. For GO BP analysis, 808 significant terms emerged, and again the results are displayed as a pie graph in Figure 7c. ClueGO analysis highlights, as the top significant term, an implication for genes in chordae embryonic development (63.64% terms per group). However, numerous terms involve metabolic processes, which is interesting in the obesogenic context, including terms implicated in the glycoprotein biosynthetic process (2.12% terms per group) and the nucleobase-containing compound metabolic process (4.55% terms per group) (Figure 7c). Lastly, 57 significant terms emerged in the GO CC analysis, and the results are displayed as a pie graph in Figure 7d. Interestingly, a strong implication concerns the nucleus (27.27% terms per group), along with endocytic vesicles (24.24% terms per group). Another compartment that seems to be highly involved is the Golgi apparatus, with 48.48% terms per group (Figure 7d).

### 3.7. Pathway Analysis of Coding and Non-Coding Transcripts Associations Highlights Specific Processes-Involvement 

DE RNAs were subjected to pathway analysis in three well renowned databases: Reactome, WikiPathways, and KEGG. Reactome analysis highlighted 53 significant pathways, as reported in Table S4. The top 20 pathways, ranked by their significance, are reported in Figure 7e. Interestingly, Wnt regulation and glycosylation seem to be the most represented terms. Indeed, Wnt signaling is represented by “signaling by Wnt”, “negative regulation of TCF-dependent signaling by Wnt ligand antagonists”, and “Wnt5A-dependant internalization of FZD2, FZD5, and ROR2”, whereas glycosylation and glycan biosynthesis are involved. 

Thirty pathways emerged from the WikiPathways database, as reported in Table S4. The first 20 pathways, ranked by their significance, are reported in Figure 7e. The Wnt signaling pathway is represented multiple times, along with in insulin signaling (normal and diabetic condition) and type 2 diabetes. KEGG analysis with g:Profiler highlighted only 1 significant pathway, also previously mentioned, which is the Wnt signaling pathway, as reported in Table S4.

### 3.8. Correlation between DE RNAs and Anhtropometric Parameters

**Figure 8:**
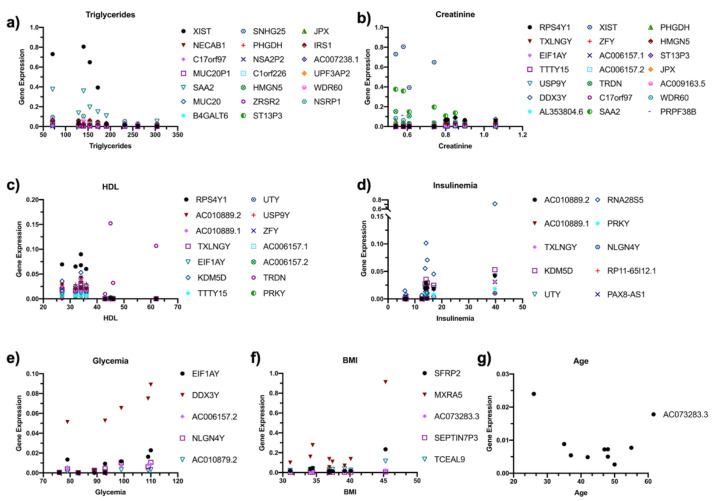
Correlation analysis of anthropometrical parameters with DE RNAs. The correlation between the expression of DE RNAs and the subject’s anthropometrical parameters was investigated, and the significantly correlated DE RNAs are displayed as correlation plots. The gene expression was correlated with (**a**) Triglycerides, (**b**) Creatinine, (**c**) HDL, (**d**) Insulinemia, (**e**) Glycemia, (**f**) BMI, and (**g**) Age.

Given the importance of lncRNAs in the biology of sex and obesity, particular attention was given to the correlation between DE RNA and anthropometric parameters, to shed new light on the role of lncRNAs in male/female obese SAT. For each patient, different parameters were considered, and their correlation with DE RNAs was displayed as a scatter plot. The full list of genes correlated with each parameter is reported in Supplementary Table S5. The highest correlation is with triglycerides and creatinine. Specifically, 20 DE RNAs (*XIST, SNHG25, JPX, NECAB1, PHGDH, IRS1, C17orf97, NSA2P2, AC007238.1, MUC20P1, C1orf226, UPF3AP2, SAA2, HMGN5, WDR60, MUC20, ZRSR2, NSRP1, B4GALT6, ST13P3*) were correlated with triglyceride levels (Figure 8a) and 21 DE RNAs (*RPS4Y1, XIST, PHGDH, TXLNGY, ZFY, HMGN5, EIF1AY, AC006157.1, ST13P3, TTTY15, AC006157.2, JPX, USP9Y, TRDN, AC009163.5, DDX3Y, C17orf97, WDR60, AL353804.6, SAA2, PRPF38B*) were correlated with creatinine (Figure 8b). Interestingly, among them, 4 out of 20 (*XIST, SNHG25, JPX, MUC20P1*) and 5 out of 21 (*XIST, TXLNGY, TTTY15, ST13P3, JPX*) were lncRNAs (Figure 8a,b). HDL correlated 14 DE RNAs (*RPS4Y1, UTY, AC010889.2, USP9Y, AC010889.1, ZFY, TXLNGY, AC006157.1, EIF1AY, AC006157.2, KDM5D, TRDN, TTTY15, PRKY*) (Figure 8c), followed by insulinemia with 10 DE RNAs (*AC010889.2, RNA28S5, AC010889.1, PRKY, TXLNGY, NLGN4Y, KDM5D, RP11-65I12.1, UTY, PAX8-AS1*) (Figure 8d), glycemia and BMI (Kg/m^2^) with 5 DE RNAs (*EIF1AY, DDX3Y, AC006157, NLGN4Y, AC010879.2 and SFRP2, MXRA5, AC073283.3, SEPTIN7P3, TCEAL9*, respectively) (Figure 8e,f respectively), and age with only AC073283.3, as correlated (Figure 8g). Five lncRNAs-DE RNAs were found correlated with HDL cholesterol and insulin levels (*AC010889.2, AC010889.1, TXLNGY, AC006157.2, TTTY15 and AC010889.2, AC010889.1, RNA28S5, TXLNGY, PAX8-AS1,* respectively) (Figure 8c,d). None of the lncRNAs-DE RNAs were related to fasting glucose, BMI, or age (Figure 8e–g). No significant correlations in DE RNAs were found when comparing gene expression to Cholesterol, LDL, AST, ALT, and white blood cell count (Table S5).

## 4. Discussion

The molecular mechanisms underlying the influence of sex- related differences on the development of obesity are still unknown. Moreover, the role of non-coding RNAs in this context has never been investigated. Transcriptional characterization of specific tissues in obese patients is of crucial relevance in highlighting the role of sex differences in obesity, fully characterizing the underlying molecular signature that leads to a different disease profile in obese men or women. To this end, the results hereby presented are an in-depth analysis of transcriptional differences occurring in the SAT of a total of 10 subjects: 5 obese women (OBF, age 41 ± 12.5 years, BMI 38.2 ± 4.6 kg/m^2^), and 5 obese men (OBM, OBM, age 42.4 ± 6.58 years, BMI 36.9 ± 3.5 kg/m^2^). Although the total data size can be considered limited, this study is still relevant in providing novel potential targets to be validated in a wider cohort. Indeed, the results highlight a subset of 51 DE RNAs, 70% of which were coding genes and 30% of which were non-coding DE RNAs. We imposed minimum |log_2_FC| of 1 and an FDR lower than 0.1 as cut-offs to differentially expressed genes in order to have a stringent significance threshold.

Through KEGG, Reactome, WikiPathways, GO enrichment analysis, and CORUM analysis, we were able to deeply investigate pathways, molecular functions, biological processes, cellular components, and protein complexes to gain more in-depth insights over pathways in which coding and non-coding genes were involved. The GO analysis for molecular function highlighted terms such as “protein demethylase activity”, “histone demethylase activity”, and “demethylase activity” as a result of the increased expression of KDM5D and UTY, and this confirms the existence of differences in epigenetic regulation in males with respect to females [50]. Moreover, the ClueGO MF analysis also highlighted MAPK activity, along with cation binding, RNA binding, and catalytic activity. Furthermore, the GO analysis for biological process highlighted metabolic processes, interesting in the obesogenic context, and protein modifications as also supported by ClueGO analysis. Pathways related to epigenetic and transcriptional regulation as well as Wnt signaling pathways, highlighted by the three renowned databases Reactome, WikiPathways, and KEGG, gives a new perspective on the mechanisms through which sex differences are present in obesity development. Indeed, although the role of Wnt signaling remains largely unexplored, evidences highlights how Wnt signaling contributes to obesity-associated metabolic dysfunction by increasing adipose tissue inflammation [51]. In our work, the two differentially expressed genes involved in this pathway are ROR2 and SFRP2, both with decreased expression in males, suggesting a decreased activation of Wnt signaling mediated by these molecules.

Using the ClinVar database, we were able to evaluate the onset of potential secondary diseases in both men and women. Along with sex-associated diseases, we also highlighted the possible onset of metabolic diseases such as diabetes mellitus type 2. Moreover, the DisGENET database allowed us to highlight specific genes related to disease conditions, divided by category. Specifically, IRS1 and PHGDH emerge as the DE RNAs causative of nutritional and metabolic associated diseases, and these are two regulators extremely relevant and characterized in diabetes, lipidemia, obesity (IRS1), and phosphoglycerate dehydrogenase deficiency (PHGDH). Furthermore, genes presented with a decreased expression in men, suggesting that female patients could be more prone to developing associated pathologies.

Given the importance of lncRNAs in sex-biology and obesity, we also focused on non-coding DE RNAs, as these could bridge the void in present day knowledge concerning the role of lncRNAs in sex-specific obesity. Using the WGCNA R package we were able to evaluate the interactions ncRNAs and coding genes, highlighting how 10 out of the 19 ncRNAs interact with 22 coding DE RNAs. These lncRNAs could thus exert their function through a modulation of the coding genes, although functional in vitro analysis is needed to validate this mechanism. Indeed, lncRNAs can act through several distinct modes, influencing gene expression at multiple levels, starting from chromatin re-arrangements and transcriptional and translational modulation [23,24,52]. Biological characterization will shed light on which processes these lncRNAs affect, which could include adipogenesis, insulin resistance, inflammatory response, or other key mechanisms present in obesity. The lncRNAs hereby reported can also be associated with the insurgence of specific diseases, such as cancer development, neurodegenerative diseases (Alzheimer’s disease and Huntington’s disease), and schizophrenia, suggesting they could also be crucial players in co-morbidities development and thus affect obesity development and prognosis. In order to see in which processes these lncRNAs could be implicated, the functional enrichment of the involved DE RNAs was analyzed to identify the specific ontology and pathway implication. As for all DE RNAs, the functional enrichment analysis highlighted a dysregulation in MAPK activity pathways as well as RNA binding and Wnt signaling, suggesting a role for lncRNAs in altering expression of the coding genes implicated in obesity through these processes. LncRNAs have been recognized as viable biomarkers for a wide number of diseases and could be used in the design of lncRNA-based therapies for obese patients [21,53].

Moreover, correlations with anthropometrical parameters highlighted how the DE RNAs expression could be influenced by specific biological parameters that differ between men and women, providing a new prospective on possible gene modulation.

## 5. Conclusions

In conclusion, even if this work is to be considered a pilot study and will need to be validated in a wider cohort, it provides a comprehensive analysis of the implications of both coding and non-coding genes in relation to obesity and sex differences. Moreover, it represents an interesting basis for the development of new patient-specific therapeutic strategies.

## Figures and Tables

**Table 1:** Number of differentially expressed coding and non-coding RNAs after transcriptome analysis, divided according to their Fold Change (FC).

	Men vs. Women	
	mRNAs	ncRNAs
Positive FC	10	9
Negative FC	22	10
Total	32	19

**Table 2:** List of DE RNAs ranked according to their absolute value of fold change (log_2_FC). The gene’s *p*.value is also reported, along with their function, as described in the STRING or UniProtKB database.

	Gene_Name	log_2_FC	*p*.Value	Function
ENSG00000129824	*RPS4Y1*	8.76	9.52 × 10^–39^	This gene encodes ribosomal protein S4, a component of the 40S subunit.
ENSG00000198692	*EIF1AY*	7.67	1.03 × 10^–15^	Encodes a protein related to eukaryotic translation initiation factor 1A (EIF1A), which may function in stabilizing the binding of the initiator Met-tRNA to 40S ribosomal subunits.
ENSG00000012817	*KDM5D*	7.65	4.55 × 10^–27^	Histone demethylase that specifically demethylates the ‘Lys-4’ of histone H3, thereby playing a central role in histone code. Role in spermatogenesis. Regulates androgen receptor (AR) transcriptional activity by demethylating H3K4me3.
ENSG00000183878	*UTY*	7.21	1.63 × 10^–23^	Male-specific histone demethylase that catalyzes trimethylated ‘Lys-27’ (H3K27me3) demethylation in histone H3.
ENSG00000114374	*USP9Y*	6.89	8.69 × 10^–31^	May function as a ubiquitin-protein or polyubiquitin hydrolase involved both in the processing of ubiquitin precursors and of ubiquitinated proteins. May therefore play an important regulatory role at the level of protein turnover by preventing degradation of proteins through the removal of conjugated ubiquitin.
ENSG00000067048	*DDX3Y*	6.35	7.37 × 10^–63^	Probable ATP-dependent RNA helicase. During immune response, may enhance IFNB1 expression via IRF3/IRF7.
ENSG00000067646	*ZFY*	5.13	2.27 × 10^–31^	Probable transcriptional activator. Binds to the consensus sequence 5′-AGGCCY-3′.
ENSG00000186439	*TRDN*	−3.79	8.54 × 10^–5^	Triadin; contributes to the regulation of luminal Ca^2+^ release via the sarcoplasmic reticulum calcium release channels RYR1 and RYR2, a key step in triggering skeletal and heart muscle contraction. Plays a role in excitation-contraction coupling in the heart and in regulating the rate of heartbeats.
ENSG00000099725	*PRKY*	3.67	6.11 × 10^–16^	Could be the product of a pseudogene. Highly similar to PRKX in the pseudo autosomal region of the X chromosome; the transcripts specific of that gene are potential candidates for nonsense-mediated decay.
ENSG00000165246	*NLGN4Y*	3.46	3.43 × 10^–12^	Putative neuronal cell surface protein involved in cell-cell-interactions.
ENSG00000123119	*NECAB1*	−2.59	0.00039	N-terminal EF-hand calcium binding protein 1; EF-hand domain containing.
ENSG00000169071	*ROR2*	−2.43	2.79 × 10^–5^	Tyrosine-protein kinase transmembrane receptor ROR2 involved in the early formation of chondrocytes. May act as a receptor for Wnt ligand WNT5A, which may result in the inhibition of WNT3A-mediated signaling.
ENSG00000145423	*SFRP2*	−2.30	0.00022	Secreted frizzled-related protein 2; modulator of Wnt signaling through direct interaction with Wnts.
ENSG00000187624	*C17orf97*	−2.13	0.00018	Protein LIAT1; may be involved in ATE1-mediated N-terminal arginylation.
ENSG00000280442	*AC010879.2*	2.022	0.00010	Unknown.
ENSG00000134339	*SAA2*	−2.01	0.00052	Serum amyloid A-2 protein; apolipoprotein of the HDL complex; belongs to the SAA family.
ENSG00000176945	*MUC20*	−1.93	0.00024	Mucin-20; may regulate MET signaling cascade. Seems to decrease hepatocyte growth factor (HGF)-induced transient MAPK activation.
ENSG00000164530	*PI16*	−1.83	0.00056	Peptidase inhibitor 16; may inhibit cardiomyocyte growth; CAP superfamily.
ENSG00000118276	*B4GALT6*	−1.76	0.00029	Beta-1,4-galactosyltransferase 6; required for the biosynthesis of glycosphingolipids; Beta 4-glycosyltransferases.
ENSG00000101825	*MXRA5*	−1.74	2.27 × 10^–5^	Matrix-remodeling-associated protein 5; in the kidney, has anti-inflammatory and anti-fibrotic properties by limiting the induction of chemokines, fibronectin, and collagen expression.
ENSG00000092621	*PHGDH*	−1.53	0.00025	D-3-phosphoglycerate dehydrogenase; catalyzes the reversible oxidation of 3-phospho-D- glycerate to 3-phosphonooxypyruvate, the first step of the phosphorylated L-serine biosynthesis pathway.
ENSG00000239887	*C1orf226*	−1.41	0.00051	Uncharacterized protein C1orf226; Chromosome 1 open reading frame 226
ENSG00000198157	*HMGN5*	−1.32	0.00054	High mobility group nucleosome-binding domain-containing protein 5; preferentially binds to euchromatin and modulates cellular transcription by counteracting linker histone-mediated chromatin compaction.
ENSG00000169249	*ZRSR2*	−1.27	2.08 × 10^–7^	U2 small nuclear ribonucleoprotein auxiliary factor 35 kDa subunit-related protein 2; pre-mRNA-binding protein required for splicing of both U2- and U12-type introns.
ENSG00000273269	*AC073283.3*	−1.25	0.00031	Unknown
ENSG00000173905	*GOLIM4*	−1.12	1.13 × 10^–5^	Golgi integral membrane protein 4; plays a role in endosome to Golgi protein trafficking.
ENSG00000169047	*IRS1*	−1.12	0.00036	Insulin receptor substrate 1; may mediate the control of various cellular processes by insulin. When phosphorylated by the insulin receptor, binds specifically to various cellular proteins containing SH2 domains.
ENSG00000261717	*AC009163.5*	−1.09	0.00017	Novel TMEM170A-CFDP1 readthrough protein
ENSG00000185222	*TCEAL9*	−1.08	0.00053	May be involved in transcriptional regulation.
ENSG00000126870	*WDR60*	−1.01	1.24 × 10^–5^	WD repeat-containing protein 60; may play a role in ciliogenesis; WD repeat domain containing.
ENSG00000126653	*NSRP1*	−1.01	7.56 × 10^–5^	Nuclear speckle splicing regulatory protein 1; RNA-binding protein that mediates pre-mRNA alternative splicing regulation
ENSG00000134186	*PRPF38B*	−1.01	2.07 × 10^–6^	Pre-mRNA-splicing factor 38B; may be required for pre-mRNA splicing

**Table 3:** nc DE RNAs emerging in OBM vs. OBF SAT, along with the number of diseases in which they are potentially implicated.

	Gene_Name	log_2_FoldChange	*p*.Value	Gene_Biotype	Number of Potential Disease Implication
ENSG00000280358	*AC010889.2*	7.99	4.36 × 10^–21^	lncRNA	N/A
ENSG00000280101	*AC010889.1*	7.85	1.12 × 10^–19^	lncRNA	7
ENSG00000131002	*TXLNGY*	7.70759269	4.37 × 10^–21^	transcribed_unprocessed_pseudogene	N/A
ENSG00000233864	*TTTY15*	7.25802788	9.28 × 10^–14^	lincRNA	20
ENSG00000277577	*AL353804.6*	−6.0911276	1.55 × 10^–9^	misc_RNA	N/A
ENSG00000229807	*XIST*	−5.3550492	5.55 × 10^–32^	lncRNA	173
ENSG00000279008	*AC006157.1*	4.35	4.38 × 10^–20^	lncRNA	7
ENSG00000226958	*RNA28S5*	4.26225122	5.56 × 10^–10^	processed_pseudogene	N/A
ENSG00000279899	*AC006157.2*	3.80	6.29 × 10^–15^	lncRNA	N/A
ENSG00000274877	*RP11-65I12.1*	2.30383605	1.66 × 10^–8^	antisense	N/A
ENSG00000224769	*MUC20P1*	−2.0972852	0.00036891	unprocessed_pseudogene	N/A
ENSG00000189223	*PAX8-AS1*	1.77943166	5.26 × 10^–6^	lncRNA	23
ENSG00000266402	*SNHG25*	−1.6343796	2.10 × 10^–5^	lncRNA	12
ENSG00000258357	*NSA2P2*	−1.4942379	0.00035201	processed_pseudogene	N/A
ENSG00000226102	*SEPTIN7P3*	−1.2425141	3.99 × 10^–5^	unprocessed_pseudogene	N/A
ENSG00000257773	*ST13P3*	−1.2340485	9.77 × 10^–6^	processed_pseudogene	N/A
ENSG00000225470	*JPX*	−1.1334427	1.24 × 10^–6^	lncRNA	24
ENSG00000231043	*AC007238.1*	−1.1074779	0.00016404	processed_pseudogene	N/A
ENSG00000214832	*UPF3AP2*	−1.0896018	8.22 × 10^–5^	transcribed_processed_pseudogene	N/A

## Data Availability

The raw data obtained from the RNA-seq analysis is deposited on the Gene Expression Omnibus repository with the accession number GSE166047.

## Supplementary Materials

The following are available online at https://www.mdpi.com/article/10.3390/jpm11050352/s1, Table S1: Clinical features of enrolled patients, Table S2: List of Primers used for Real-Time PCR validation, Table S3: Enrichment analysis of DE RNAs as obtained with g:Profiler. Terms were ranked according to their Fold Change, and a Bonferroni–Hochberg FDR of 0.05 was used as the threshold. The full list of significant pathways (adjusted *p*.value < 0.05) is reported, Table S4: Enrichment analysis of DE RNAs correlated with nc DE RNAs as obtained with g:Profiler. Terms were ranked according to their Fold Change and a Bonferroni–Hochberg FDR of 0.05 was used as the threshold. The full list of significant pathways (adjusted *p*.value < 0.05) is reported, Table S5: Correlation analysis of anthropometrical parameters with DE RNAs. The full list of genes correlated with each parameter is reported, and a white cell indicates non-significant correlations, a blue cell terms with * *p* < 0.05, a green cell terms with ** *p* < 0.01, and an orange cell terms with *** *p* < 0.001.
