# Supplementary material for: RNA-seq Characterization of Sex-Differences in Adipose Tissue of Obesity Affected Patients: Computational Analysis of Differentially Expressed Coding and Non-Coding RNAs
Source: J Pers Med. 2021 Apr 28;11(5):352. doi: 10.3390/jpm11050352 (PMC8145808; doi:10.3390/jpm11050352)
Supplement: Supplementary file 1 [file jpm-11-00352-s001.zip › Rey et al_Supplementary/Supplementary_Table_S2.pdf]

| | |
|---|---|
| 18S-FW | AGTACGCAGGGCCGGTACAGTGAAACTGCG |
| 18S-REV | CGGGTTGGTTTTGATCTGATAAATGCACGC |
| TTTY15-FW | GGACCGGGAGATAGGAGTGT |
| TTTY15-REV | CACGGACTCCAGGTGATGAG |
| UTY-FW | GCACCACTGGTTTTGTAGCTG |
| UTY-REV | GCACTGTGTCCAGTTGCTTG |
| KDM5D-FW | TGGTGTTTGTAGTGCTCTGTGA |
| KDM5D-REV | TGGTGTTTGTAGTGCTCTGTGA |
| XIST-FW | TAGGTGGAGATGGGGCATGA |
| XIST-REV | GCCCAGTGGTAGTGAGCTTT |
| ROR2-FW | TAACCAGCACAAACAGGCCA |
| ROR2-REV | ATGAACCTCACCGCAGACAG |
